# Digital natives aren’t concerned much about privacy, or are they?

**DOI:** 10.1515/icom-2022-0041

**Published:** 2023-03-15

**Authors:** Edith Maier, Michael Doerk, Ulrich Reimer, Matthias Baldauf

**Affiliations:** Eastern Switzerland University of Applied Sciences, Institute for Information and Process Management, St. Gallen, Switzerland; Lucerne University of Applied Sciences, Institute of Social Pedagogy and Education, Lucerne, Switzerland

**Keywords:** data protection, privacy, privacy by design, privacy paradox, voice assistants

## Abstract

Voice assistants have become embedded in people’s private spaces and domestic lives where they gather enormous amounts of personal information which is why they evoke serious privacy concerns. The paper reports the findings from a mixed-method study with 65 digital natives, their attitudes to privacy and actual and intended behaviour in privacy-sensitive situations and contexts. It also presents their recommendations to governments or organisations with regard to protecting their data. The results show that the majority are concerned about privacy but are willing to disclose personal data if the benefits outweigh the risks. The prevailing attitude is one characterised by uncertainty about what happens with their data, powerlessness about controlling their use, mistrust in big tech companies and uneasiness about the lack of transparency. Few take steps to self-manage their privacy, but rely on the government to take measures at the political and regulatory level. The respondents, however, show scant awareness of existing or planned legislation such as the GDPR and the Digital Services Act, respectively. A few participants are anxious to defend the analogue world and limit digitalization in general which in their opinion only opens the gate to surveillance and misuse.

## 1 Introduction

In a 4-year project (2020–2023) funded by the Swiss National Science Foundation, a team of researchers with expertise in human-computer interaction, home automation, digital services, data science, and behavioural economics has been investigating how the presence of voice assistants affects people’s domestic lives, routines and values. Like chatbots voice assistants interact with humans using natural language, but they are not domain-specific and as opposed to most chatbots, voice assistants are speech-based and can be integrated into many devices such as smartphones, smart speakers, (e.g. Alexa or Google Assistant), service and social robots or cars. With their voice assistants users can perform various tasks, control other devices such as lights and enjoy third party services. As voice assistants are embedded in people’s lives, they gather enormous amounts of personal information which is why they evoke serious privacy concerns regarding the collection, use and storage of personal user data by large tech companies.

The focus of this paper is on attitudes to privacy and intended behaviour in privacy-sensitive situations and contexts. It also discusses ideas and recommendations addressed to governments, organisations or companies about the measures they might undertake to protect people’s personal data. It extends previous work [1, 2] based on empirical data collected from two groups in 2021: on the one hand a group of volunteers of different ages and backgrounds who recorded their daily activities and experiences with voice assistants (referred to as *in-home study*) and on the other hand, a group of students in their early twenties who engaged with voice assistants as part of their course work (referred to as *student group*).

In the data analysis, privacy and data protection emerged as key issues. Participants of both the in-home study and the student group agreed that privacy was important and should be protected. One of the surprising results of the previous research was that the digital natives – the generation of people who grew up in the era of ubiquitous technology [3] – proved to be even more concerned about protecting their privacy than digital immigrants and tended to be more aware of the potential that breaches of privacy and security might entail. This appeared to contradict findings we had come across in other studies such as [4] or [5]. At the same time, many responses revealed a high degree of ambivalence, resignation or even cynicism, feelings which we decided to explore in more depth.

For the spring semester 2022, we adjusted the course content and requirements to obtain a more fine-grained picture of the attitudes held by digital natives. The new group of students was informed about the rationale behind the focus on privacy and data protection, without providing any detail about previous findings, however. We developed a series of privacy-sensitive scenarios to elicit responses as to the potential risks and outcomes associated with the different scenarios. We also wanted to find out if participants distinguished between different types of data when it came to assessing risk and if the purpose to which their data would be used made a difference. Besides, we decided to broaden the topic to include all kinds of speech-based or conversational agents and thus move away from the exclusive focus on voice assistants, which by many were only seen as the smart speakers sitting in their homes instead of as assistants they could connect to anywhere via their mobile devices.

Furthermore, given the limits of self-managing privacy (see [6]) we encouraged the students to suggest measures that could be taken at the political, regulatory and organisational level to protect one’s personal data and mitigate the risks to privacy more effectively. The aim was to gain valuable insights for policymakers. Also, we wanted to find potential solutions that might help span the gap between users’ attitudes to privacy and intended and actual behaviour. According to the conclusions drawn in the surveys conducted by [7, 8], such practical elements are missing in the current literature on privacy.

As shown in many previous studies, attitudes are poor predictors of behaviour (e.g. [6, 9]). The empirical data show that few actually take steps to self-manage privacy e.g. by adjusting settings or encrypting their data. Instead most people appear resigned and simply surrender to (potential) violations to their privacy. Some users are quite aware of the contradictions between their privacy-related attitudes and their actual behaviour in other realms of their lives. For example, they point out that by accepting loyalty cards from shops they are willing to disclose the content of their shopping baskets in return for discounts. And many just accept all cookies when they want to use a particular web-based service, either because they are in a hurry or cannot be bothered.

Therefore, the risks associated with voice assistants also apply to smart technologies in general including wearables, smartphones or large language models such as ChatGPT. However, because of the convenience offered by voice assistants esp. with regard to completing tasks such as turning lights on and off or setting alarms without the need to type, read or hold a device, people may be more “tempted” to use them and disregard potential risks [10]. The benefits therefore may give rise to a new set of risks that can make people vulnerable to violations of their privacy [11].

In the following sections we first discuss findings from other studies related to the use of voice assistants and how they influence people’s activities, norms and domestic lives. After all, the home is considered a private space which is then invaded by devices that constantly listen and may transmit data to the outside world. In Section 3, we explain how we proceeded methodologically with regard to collecting and analysing and interpreting the data. In Section 4, we present the results where we distinguish between responses to the different scenarios and the suggestions addressed to governments and organisations. This leads us to embed the question “What can be done?” in a wider context and look at privacy-protecting measures taken or to be taken at political-economic and legislative levels (in Section 5). Section 6 concludes the paper and gives an outlook.

## 2 Related work

With the rising popularity of conversational agents such as smart speakers or chatbots, the Internet of Things (IoT) has finally reached people’s homes and has become part of everyday life. Conversational agents are software systems designed to interact with humans using natural language. They are equipped with technologies that can, and often do, monitor activities to collect, record and transfer all kinds of data to an external information domain. They have been identified as the first contact or touch points that, once introduced into a home, stimulate users to expand them with further functionalities by connecting them with other devices like smart phones, cars, televisions, microwaves, fridges, and even toothbrushes [12, 13].

A range of relevant studies on the use and impact of technology in everyday life have emerged within different disciplines. Previous studies on technology adoption in private households were conducted with an eye to technology acceptance [14, 15], but also looked at the risks and societal consequences that might be associated with conversation-based technologies [16]. The threats which these devices pose to people’s privacy, figure most prominent among the risks discussed [17–19].

Privacy risks have shown to have a dampening effect on people’s adoption and use of voice assistants (e.g. [10]). Especially news items related to leaks of personal details or their unintended transmission to third parties, have made individuals more sensitive to potential risks. As a result, tech companies such as Google or Amazon have taken steps such as voice printing to identify the user of the device and prevent it from detailing personal information. Still, many users are not convinced and therefore avoid talking about sensitive topics or refrain from making payments with a voice assistant (e.g. [20]).

### 2.1 The privacy challenge

Privacy is a multi-faceted concept and has different dimensions, e.g. security, secrecy, autonomy and control. Privacy is valuable because it puts limitations on power, demonstrates respect towards others, enables people to manage their reputations, is a prerequisite for establishing and maintaining trust and candour in relationships and is essential for the control over one’s life [6]. Privacy is also about modulating boundaries between public and private spaces and controlling the data flow between the two. According to Weinberger et al. [21] social norms around privacy dictate that in some circumstances we are not allowed to notice or eavesdrop on a conversation, and in others we can.

Solove [6] and Véliz [9] also consider privacy to be essential for freedom of thought and speech as well as social and political activities. It allows people to change and have second chances, protects their intimacy and it frees persons from having to explain and justify themselves. According to Solove [6] privacy is a constituent element of a free and democratic society, an opinion which is shared by others such as Schwartz [22] or Véliz [9].

When it comes to assessing people’s privacy attitudes, many researchers use the Privacy Segmentation Index introduced by Westin [23]. It has been widely used to categorize such attitudes and to make longitudinal comparisons [24, 25]. Westin’s Index categorizes individuals into three privacy groups:

1. *Unconcerned* – those who give privacy little thought
2. *Pragmatists* – those who worry about threats to privacy but believe that reasonable safeguards are in place or can be created
3. *Fundamentalists* – those with high privacy concerns and high distrust in government, business, and technology

One might expect that people’s attitudes would have a significant impact on their behaviour. Previous research, however, has failed to establish a robust correlation between the Westin categories and actual or intended behaviour. Numerous studies have documented an attitude-behaviour dichotomy (also referred to as the Privacy Paradox), in which participants’ privacy-related attitudes do not coincide with their behaviour (see e.g. [8, 17]).

Woodruff et al. [26] explored the connection between the Westin categories and individuals’ responses to the consequences of privacy behaviour. For this purpose they conducted a survey of 884 Amazon Mechanical Turk participants to investigate the relationship between the Westin Privacy Segmentation Index and attitudes and behavioural intentions for both privacy-sensitive scenarios and their possible consequences. Participants were asked to imagine divulging or not divulging personal data related to their financial situation, health or location and the context of the disclosures. This may include the party to whom the information was to be disclosed, online versus offline, whether or not the information was anonymized, and when or if the information would be deleted. Besides, the consequences of the disclosure as well as a range of positive and negative outcomes with different financial, health, social, and other impacts could be specified. The results showed a lack of correlation between the Westin categories, intended behaviour and possible outcomes or consequences.

### 2.2 Theoretical attempts at explaining the privacy paradox

According to Gerber, Gerber and Volkamer [8] there is strong evidence that the so-called “privacy calculus model” is one of the best predictors for both people’s intended and actual disclosure of personal information and one of the most-established explanations for the privacy paradox. It was originally put forward by Culnan and Armstrong [27] who argued that the intention to disclose personal information is based on a rational risk-benefit calculation as perceived benefits are weighed against risk probability. If perceived benefits outweigh risks, people would be willing to divulge such information in exchange for social or economic benefit.

On the other hand it is argued that situational cues, cognitive biases and social norms influence privacy decisions and risk assessments rather than generic attitudes. According to the Theory of Bounded Rationality [28], for instance, individuals tend to be satisfied with a solution that is good enough but not optimal due to cognitive limitations and the limits of available time. The study of Kehr et al. [29]; for instance, has shown that privacy decisions are driven not only by general dispositions such as institutional trust or general attitudes, but also by situation-specific assessment of risks and benefits as well as affect-based heuristics which are often subconscious. This explanation largely coincides with the “behaviour distortion argument” inspired by the work of Kahnemann, who distinguishes between slow and fast thinking [30].

Thus, we can distinguish between two types of arguments that try to explain the privacy paradox: the “behaviour valuation argument” and the “behaviour distortion argument” (see e.g. [6]). The first contends behaviour is the best metric to evaluate how people actually value privacy. Since people are willing to disclose personal data for “free” goods or services, it is concluded that they ascribe a low value to privacy. According to the second, i.e. the behaviour distortion argument, people’s behaviour is not an accurate metric of preferences because behaviour is distorted by biases and heuristics, manipulation and framing (see e.g. [7, 21, 31]).

However, the value of both arguments can be and has been called into question (see e.g. [6, 9]). Just because people are willing to disclose personal data for little or nothing in return, we cannot conclude that they ascribe a low value to privacy. Actually, we live in an age where it is nearly impossible not to disclose personal data if one wants to participate in social life and engage in economic activities. People constantly make risk assessments when they trade their personal data in exchange for gaining access to information or services important to them. In a comprehensive empirical study by Kesan et al. [32] on data privacy, trust and consumer autonomy, more than 80% of respondents said that they had at least once provided information online when they wished that they did not have to do so. As Mai [33] puts it:

People reveal personal information consciously or unconsciously, willingly or unwillingly, as they perform everyday activities: shopping for groceries, communicating with family members, paying taxes, reading the news, listening to music, reading e-books, purchasing gasoline, exchanging e-mails, sharing photos, and so on. In addition, many people choose to reveal information about their private lives on social networking sites.

Besides, many are not aware that the value of data (as well as the inferable information and potential risk) is increased during the aggregation of different bits of data from different sources. Other reasons given for the dichotomy include the fact that Westin’s categories only measure general attitudes, while behaviour is context-specific and that individuals may perform privacy risk assessments but choose the most viable or convenient options, even if they are not in accordance with their privacy preferences (for an overview, see [8]).

### 2.3 Going beyond the privacy paradox

If the privacy paradox is a myth as claimed by Solove [6], how can the contradictions between people’s attitudes to privacy and actual or intended behaviour be explained? Many users report feelings of helplessness and powerlessness when faced with the challenge involved in protecting one’s personal data, which may translate in so-called “privacy cynicism” as discussed by Lutz et al. [34]. They highlight the multidimensionality of the construct which encompasses aspects such as uncertainty, powerlessness, mistrust and resignation. How to cope with these sentiments?

A recent paper [35] calls for designers to become more involved with the development of trust, privacy and security in the emerging technological landscape of the voice-enabled Internet. They used films to speculate on the true nature of voice assistants questioning the idea that they are our friends rather than devices which ultimately try to sell you something. Seymour and Van Kleek [36] also voice concerns about the social nature of conversational agents which might unconsciously shape our interactions with them. Their survey of 500 users has shown that people develop social relationships with voice assistants that are linked to perceptions of trust in devices, trust in manufacturers, and anthropomorphism of those devices. Seymour and Van Kleek conclude that

The transition to conversational interfaces that allow for easy and natural interaction with devices represents a profound shift in the nature of the systems we interact with towards the increasingly social. This shift brings with it a variety of new or exacerbated ethical concerns that designers of voice interfaces need to consider when designing future products.

They argue that developers should steer their designs away from potential ethical problems. This coincides with Véliz’ argument that data collection carries with it a moral responsibility and a duty of care towards data subjects [37]:

Data can tell on us: whether we are thinking about changing jobs, whether we are planning to have children, whether we might be thinking of divorcing, whether we might be considering having an abortion. Data can harm people.

## 3 Methodological approach

As mentioned in the Introduction, the rationale for this paper is largely derived from the findings of the empirical data discussed in Maier et al. [1] and Riss et al. [2]. We found the results intriguing because they did not coincide with our expectations based on relevant literature, namely that digital natives were less concerned about protecting their personal data.

The participants for this study therefore only comprised digital natives: students mostly in their early twenties, living on their own or with fellow students and rather than volunteering, they had to engage with a voice assistant as part of their study requirements for an interdisciplinary course on resource management over a period of 13 weeks.

As far as the gender of participants was concerned, we had a fairly even distribution, namely 28 female and 37 male students. Students reported that they most frequently used smartphones and notebooks or tablets. Quite a few (14) had voice-enabled smart television sets and navigation devices. Almost half of the participants were enrolled in social studies, followed by 15 in technology, 11 in architecture, and 4 each in computer science and business administration. Only one student was enrolled in the design & arts department. No correlations between age, gender, and academic discipline could be found.

**Figure 1:**
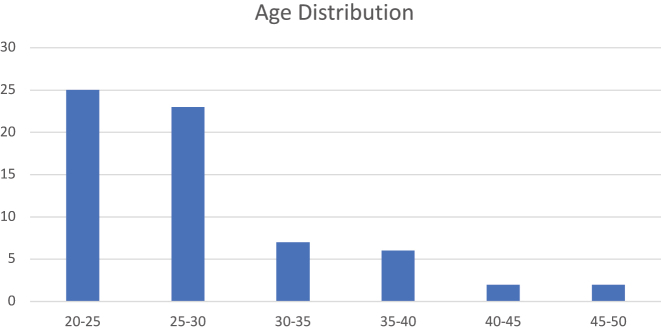
Age distribution of student group participants.

As can be seen in Figure 1, most participants were between 20 and 30 years old. The youngest participant was 21 and the oldest was 49 years old and might therefore be considered a digital immigrant rather than a native.

### 3.1 Data collection

As a result of the findings discussed previously in Maier et al. [1] and Riss et al. [2], we included questions to obtain a more detailed and quantifiable overview of students’ attitudes to privacy. For this purpose, we adopted the Westin categories but slightly adapted the phrasing of the statements related to the pragmatist and fundamentalist categories (see Table 1). Rather than referring to safeguards to protect one’s privacy we referred to the benefits outweighing the risks involved in using a conversational agent so as to better match the assumptions of the privacy calculus theory. As far as the fundamentalist category was concerned, we put the emphasis less on distrust of government or technology, but rephrased the question in a way to ascertain who was very concerned about the threats to privacy in general. We also designed scenarios to explore individuals’ perceptions of privacy in specific contexts or situations.

**Table 1:** Frequency of use correlated with importance attributed to privacy when using voice assistant.

How important do you consider		Unconcerned (“not important,	Privacy concerns (“I do	Critical (“I consider privacy	Total
privacy and data protection		I don’t really care where	care but when the	very important therefore I	
when using a voice assistant (VA)?		data is stored or processed	benefits outweigh the	deliberately restrict the use of	
		provided the VA works”)	risks, I use the VA”)	the VA”)	
**How often do you use the VA?**	Several times a day	5	28	5	38
	Several times a week	1	6	0	7
	Rarely	4	6	7	17
**Total of column**		**10**	**40**	**12**	**62**

The scenarios were inspired by Woodruff et al. [26] and were supplemented with scenarios specifically relevant to the use of voice assistants. Furthermore, to obtain information about people’s willingness to pay for privacy-friendly online services we included potential tradeoffs in two scenarios (cp. [5, 38]):

1. You can join an insurance plan which offers you the option of putting all of your health data in a unified healthcare database. All hospital staff and emergency personnel will have access to these records without your consent.
2. The company you are working for would like to track your click behaviour to obtain general information about stress among their personnel. They intend to offer a program to alleviate stress later on.
3. You want to access a news website with your voice assistant which offers you the option to receive free information tailored to your interests, background, age etc. based on your input, or to pay a subscription fee of 10 Swiss francs per month if you prefer “neutral” information.
4. You can download a free smartphone app that automatically collects data on your exercise routes, sleep habits, and occasionally asks you how you feel. It analyses and visualizes the data and posts it publicly online without your name.
5. Your home insurance offers you a reduction of your premium of 50 Swiss francs in return for receiving smart-home data (temperature, lighting etc.) which are collected by your voice assistant. According to the insurance, this data should help protect you against burglaries.
6. You come across a vacancy ad for your dream job. When filling in the online form you’re asked to provide your login data to your social media accounts to help the HR unit find out if you match their corporate culture. Once the decision is taken, your data will be deleted.

To elicit participants’ attitudes to these different scenarios as well as get an idea of how they assessed the risks or potential outcomes if they performed a particular action or took up the offer, we also included questions as originally posed by Woodruff et al. [26]:

– How likely would you perform a given action or take up a specific offer (e.g. disclose personal health data which can then be used e.g. in case of an emergency, or disclose home surveillance data in return for a lower insurance premium)?
– How well do you think you can foresee what might happen?
– How risky do you feel it would be to disclose such information?
– What might be the consequences your actions have?

We considered these questions to best correspond to the privacy calculus model, which could be expected to predict people’s intended and actual disclosure of personal information (see [8]).

We also wanted to elicit answers to possible privacy-protective measures at the individual, the organizational, regulatory as well as political levels. This is why at the end of the semester students were asked the following questions:

– In your opinion, how could your government help people protect their privacy in a digital world?
– What sort of advice would you give to your company or your university so as to mitigate the concerns held by their customers, employees or students with regard to data protection or privacy?

With these questions we intended to add a practical element to our research and gain insights that might be relevant to policy makers as well as organisations.

### 3.2 Data analysis

Data was captured from 65 participants1A total of 65 participants enrolled in the course. However, not all of them replied to all the questions which is why the total can vary slightly. from different disciplines including social studies, architecture, design and arts, technology. A qualitative data analysis was conducted for the free-text comments to both the scenarios as well as the open questions. We defined a series of high-level categories to guide our analysis. These included data protection, privacy, smart home, transparency, digital competence, surveillance, targeted advertising as well as privacy cynicism. The last category was adopted from Lutz et al. [34] and encompasses feelings of uncertainty, powerlessness, mistrust and resignation. These categories were used to efficiently label data and speed up analysis [39].

For the quantitative analysis we used the statistical tool SPSS. Apart from analysing demographic data such as age, gender, and academic discipline, we computed the relation between the frequency of using a voice assistant and the importance users attributed to privacy to find out if there were any contradictions between attitudes and actual use (see Table 1). We also compared people’s responses to the different scenarios to identify if type of data, setting or potential benefits had an impact on people’s risk assessments or the likelihood of taking a particular action (see Table 2).

**Table 2:** Results regarding risk assessments for different scenarios.

	How likely would youperform a given action or takeup a specific offer?(0–6, 6 = very likely)	How well do you thinkyou can foresee whatmight happen?(0–6, 6 = very well)	How risky do you feelit would be to disclosesuch information?(0–6, 6 = very risky)
1. Unified health database	3.44	3.33	4.82
2. Click behavior at work for stress study	2.98	3.47	4.31
3. Personalized news tracking or pay	2.87	3.07	4.67
4. Free health tracking app with published data	2.82	3.09	5.11
5. Reduction of insurance premium based on	2.31	3.02	4.98
smart home data			
6. Temporary open social media account access	1.65	3.29	5.56
for a job			

## 4 Results

The results have been divided into general attitudes to privacy, the risks associated with the different scenarios and the responses to the open questions, i.e. the privacy-protecting measures to be taken at the political-economic level. Privacy self-management measures have also been included even though they did not form part of the questionnaire.

### 4.1 General attitudes to privacy

As explained in the Introduction we decided to conduct a more systematic and in-depth study of the attitudes of digital natives towards privacy and cast more light on the gap between attitudes and actual and intended behaviour. In line with the Westin categories [23], we can distinguish between three different attitudes regarding privacy:

1. *Unconcerned* about privacy and/or unrestricted use of voice assistant
2. *Privacy concerns*, but willing to engage with voice assistant if they see an added value in its use (Westin category of pragmatists)
3. *Critical*, very restricted use of voice assistant (Westin category of fundamentalists)

Overall, most people care about privacy as shown in Table 1. Only ten respondents are unconcerned about data protection and just want the voice assistant to function properly, whereas 52 out of 62 respondents are concerned to different degrees. Most are pragmatists who care about privacy but weigh the potential pros and cons of sharing information and are ready to make trade-offs if the benefits are big enough. Twelve respondents harbour serious concerns with regard to their privacy, but nevertheless use the voice assistant rarely or several times a day.

### 4.2 Responses to scenarios

As shown in Table 1 there is little correlation between attitudes to privacy as defined by Westin’s categories and intended and actual behaviour. Even out of those who are very concerned about their privacy, five respondents do use the voice assistant several times a day!

To cast more light onto these seeming contradictions and obtain a more differentiated view we designed scenarios to explore individuals’ perceptions of and attitudes to privacy in specific situations and contexts (see Section 2). Table 2 provides an overview of the responses about how risky the students regarded the different scenarios and how likely they would perform a particular action or take up a particular offer.

As can be seen in Table 2, making one’s private social media account accessible to an outside party, was considered least acceptable. Hardly anyone would be likely to hand over their login data even if this meant forgoing their dream job. Similarly, respondents would be unlikely to divulge their smart-home data in return for (rather modest) economic advantages. Overall, people’s risk assessments are negatively correlated with the likelihood with which they would perform an action or take up an offer. The only action where respondents might consider revealing their personal data is Scenario 1, where their health data would be made accessible to health professionals. In case of an accident or a seizure, this could be life-saving. But even there, most respondents have reservations. As far as being able to foresee possible outcomes or consequences of disclosing personal data, the majority appears to be uncertain. In the following we look at people’s reactions to the different scenarios in more detail.

1. **You can join an insurance plan which offers you the option of putting all of your health data into a unified healthcare database. All hospital staff and emergency personnel will have access to these records without your consent.**

Even though health data are generally considered sensitive, the reactions to this scenario tended to be more positive, perhaps because no obvious commercial interests seemed to be involved. Besides, in case of an accident it would be very helpful for the ambulance team to have access to a person’s health data such as blood group, medication or allergies. Nevertheless, many replies reflected fears of potential abuse and a mistrust of the data-collecting organisation. Besides, most want to stay in control of their data and determine who has access.

Since it is a private company I am sceptical. The information could simply be sold to other companies, which then might send me targeted advertising and offers.

My confidential data could end up in the hands of the insurance companies. Based on my medical record, individual price adjustments could be made to attract or entice me away as a customer.

The insurance company has access to my health data [and] can draw conclusions from it in combination with other data.

2. **The company you are working for would like to track your click behaviour to obtain general information about stress among their personnel. They intend to offer a program to alleviate stress later on.**

Tracking an employee’s clicking patterns on the keyboard to learn more about stress at work, was seen as slightly less risky. Still, many participants stated that they would feel under surveillance and would not welcome it. Besides, some argued, anti-stress programmes could be offered without having recourse to click data, which might also be used for other purposes, such as drawing conclusions about an employee’s productivity.

Employers could monitor how productive the employee is.

The data could be associated with me personally and end up not being anonymized after all.

3. **You want to access a news website with your voice assistant which offers you the option to receive free information tailored to your interests, background, age etc. based on your input or to pay a subscription fee of 10 Swiss francs per month if you prefer “neutral” information.**

The reactions to this scenario were slightly less critical but still most respondents were reluctant to reveal information about their background or interests.

Even if I don’t give my name, my mobile phone number is registered and can be traced back to me.

I would be uncertain as to what the data is needed and used for. That would make me suspicious.

Some participants pointed out that information tailored to one’s interests would actually reinforce the presence of filter bubbles.

I don’t want personalised messages. We are in a bubble enough as it is.

Reading only personalised feeds is not good for forming [one’s own] opinion.

4. **You can download a free smartphone app that automatically collects data on your exercise routes, sleep habits, and occasionally asks you how you feel. It analyses, visualizes the data and posts it publicly online without your name.**

The transfer of data to the company was generally regarded as a high risk because of possible misuse.

By regularly tracking my jogging route […] the information could be misused for a burglary, for example.

My training routes would then be public. It’s never good if others always know where you are or what you eat.

My data could be misused. By analysing photos or based on GPS information, for example, conclusions could be drawn about my place of residence, as well as.

5. **Your home insurance offers you a reduction of your premium of 50 Swiss francs in return for receiving smart-home data (temperature, lighting etc.), which are collected by your voice assistant. According to the insurance, this data should help protect you against burglaries.**

An increasing number of people use their voice assistant to control appliances such as lights or thermostats. But the majority would be unlikely to grant access to their smart-home data because they consider it risky to disclose such information and/or do not trust the insurance company:

This is sensitive data that I do not want to disclose – for the sake of privacy.

I could be proven partly responsible [for some damage] and the insurance company might refuse to pay.

Insurance companies often look for loopholes where they can save money.

Besides, many respondents argued that transmitting such data to the insurance company would allow it to surveil one’s private environment:

I would always feel under surveillance in my own flat.

6. **You come across a vacancy ad for your dream job. When filling in the online form you’re asked to provide your login data to your social media accounts to help the HR unit find out if you match their corporate culture. Once the decision is taken, your data will be deleted.**

Giving a potential employer temporary access to one’s social media account was considered a no-go by the majority. Many expected a misuse by the employer, despite the employer’s assurances that the login data would be deleted. Revealing one’s password, in particular, was seen as a security risk.

I would never do that. It’s nobody’s business what I do in my private life. No job in the world is worth it.

My […] employer has absolutely no business in my private social networks. For me, that would be crossing a red line.

For those who accepted the services or benefits offered in the various scenarios, better service quality or improved customer experience were the main reasons given for disclosing personal information. One person expressed the opinion that the existing data protection as it exists was sufficient.

Overall, we can distinguish between concerns that are specific to a particular scenario and more generic concerns. An example for a specific concern is the statement that personalising news would reinforce “bubbles”, i.e. people would no longer be exposed to different opinions. Under the latter type of concern we can subsume the fear of surveillance and a general uneasiness about an invasion of one’s private space. As far as the potential misuse of one’s personal data is concerned, people are afraid that their data might be sold to advertising companies without their knowledge. This wide-spread fear of misuse is closely connected with the lack of transparency with regard to the use, processing and storage of personal data. Also, most people doubt that anonymization really works despite the assurances of the data-collecting organisation.

### 4.3 Responses to open questions

Out of 65 participants, 51 replied to the questions regarding possible privacy-protective measures to be taken by governments or organisations. Whereas a few responses were quite extensive and included several suggestions, others were short and came up with just one particular measure or statement. Apart from suggesting measures at the legislative and regulatory level, quite a few participants recommended ways to protect one’s privacy at the individual level.

#### 4.3.1 Privacy-protecting measures at the individual level

The self-management measures proposed are as follows:

– reading privacy policies more carefully
– deleting one’s search history
– divulge as little personal data as possible and only accept technically essential cookies
– calibrating one’s privacy settings on websites or apps
– opting out

The last option occurred only once and was phrased in the form of an exhortation:

History has shown that data are sold and misused. Therefore let’s walk in the forest, dance, sing and laugh. No need to install any cameras, or mikes in our homes or implant chips in our bodies.

Another respondent was somewhat less radical, but encouraged people to restrict their activities in the digital world:

Just don’t buy any voice assistants such as Alexa and spend less time with your mobiles, because life in the real world is more important. Not everything that’s digital is better.

Still, the effort to self-manage one’s data protection such as having to deal with cookie settings on every page is considered (too) high. Also, privacy policies are regarded as (too) complicated, incomprehensible and time-consuming or cumbersome to read. They often feel tricked into accepting terms and conditions without knowing or being informed about possible consequences. So-called “dark patterns” are a recurrent theme in the responses. They refer to deceptive design patterns or tricks used in websites and apps that make you do things that you did not mean to, like buying or signing up for something.

Overall, the students spend very little time on individual privacy-protecting measures such as using a VPN client or rejecting unnecessary cookies as can be seen in Table 3. To the question “How much time do you spend on data protection when using a voice-enabled device?”, more than half of the respondents (53.9%) stated a few seconds. This may be explained by the fact that a user just clicks on “accept” or “approve” for every security request, or that defaults may have been set in advance in line with a user’s preferences. According to their own statements, 42.9% of the students take between 30 s and one minute. Only 3.2% or two of the 63 students take more than one minute for data protection.

**Table 3:** Time spent by participants on privacy-protecting measures.

How much time do you spend on average for privacy-protecting measures when using a voice-enabled device?		
	Number of students	Percentage
No time to a few seconds (e.g. you just accept all cookies)	34	53.9%
Between half a minute and just under a minute	27	42.9%
More than a minute	2	3.2%
Total	63	100.0%

#### 4.3.2 Privacy-protecting measures to be taken at the political-economic level

Most participants suggest that privacy-protective measures are to be taken at the political-economic level because they feel discouraged from self-managing their privacy. They feel forced into a take-it-or-leave-it choice if they want to use digital services or search for information in the digital world. Apart from the last two measures proposed, the students’ suggestions largely coincide with measures stipulated by existing data protection legislation such as the GDPR or legislation planned at EU level, in particular the DSA (Digital Services Act).

Measures proposed include:

– Privacy policies should be clear, easy to understand, avoid legal jargon and be phrased in simple language.
– The transfer of personal data to third parties should be prohibited or at least restricted.
– It must be made transparent how data is used, processed and stored.
– Personal data may be collected for use only if informed consent is obtained.
– Data access should be restricted to the context of user activities (e.g. online-searching).
– Companies should be made to comply with existing regulations such as GDPR e.g. by establishing (and funding!) independent auditors.
– In case of non-compliance and privacy violations sanctions should be imposed.
– Outlaw “dark patterns”!
– Organise public campaigns to raise awareness about the importance of privacy and data protection.
– Offer courses to increase people’s know-how and competence in how to protect their privacy.

The last two recommendations addressed to both governments and companies reflect respondents’ belief that *promoting digital literacy* might help tackle the problem. “More knowledge leads to more security”, as one respondent puts it.

I believe that the state should educate people […]. For example, a school subject ought to teach children how to protect their privacy. The same applies to older people. The state could commission Pro Senectute [a Swiss association representing the elderly] to inform them about this topic.

A checklist with to do’s and dont’s [would be helpful], which also should include examples of how to protect one’s privacy when dealing with voice assistants. Besides, a sort of quality label (comparable to TÜV [motor vehicle inspection]) might give users some measure of certainty that the voice assistant is trustworthy.

The demand for *more transparency* is shared by a majority of respondents. Laws and regulations should be put in place that require providers of online services to be transparent about how they use and process data and prevent them from collecting and/or selling sensitive personal data. Above all, tricking users into divulging personal data or accepting unnecessary cookies should be outlawed, most students argue. Many resent that they constantly have to agree to terms and conditions before they can use a device, website or app. Instead, defaults should be set in a way that guarantees the highest possible level of privacy.

In my opinion the state is responsible for protecting the data of its citizens. And clear regulations have to make sure that data may only be passed on if the user knows about it and not because it is allowed in the small print.

…We need detailed information about what happens to one’s data. If a company that collects data is obliged to enable the tracking of one’s personal data this will not stop any internal use of personal data but may restrict its transmission to third parties. The legislator could also require that data is only stored in anonymous form.

Some respondents suggest that the government should create a *dedicated public authority* to enforce privacy regulations. At the same time quite a few have doubts as to whether the advanced technological know-how required for such a task actually is available inside the government. Besides, quite a few feel that legislation always tends to lag behind and has not kept pace with the advances of technology. According to one respondent there will always be loopholes and grey zones before the law catches up or comes into force.

Overall, most respondents appear to trust their own, i.e. the Swiss government, to protect their data whilst at the same time realizing that efficient data protection requires *global solutions* since the digital world does not recognise any state boundaries. Furthermore, most big tech companies are based in the United States and are therefore not subject to Swiss legislation. In many statements respondents express their mistrust of big tech whilst at the same time feeling powerless and helpless. Most are resigned to forfeit part of their privacy in return for being part of digital society. Even those who harbour serious concerns are not ready to opt out altogether. As mentioned before these feelings can be subsumed under the concept of “privacy cynicism”, which emerges as a predominant theme in the replies. Some even appear to have given up hope that things will change:

It would be nice if it were transparent (even for the layman) to know which data has been collected about oneself and to be able to delete it – but utopian.

As far as possible measures to be taken by companies or organisations are concerned, the replies fall into similar categories. The advice or recommendations given include the *call for more transparency* about the use of personal data, the organisation of classes or courses to increase students’ or employees’ digital literacy, and compliance with existing data protection laws. Transparency including reporting incidents of data leaks is seen as a prerequisite for trusting an organization to handle one’s data in a responsible way. Besides, companies and organisations should communicate clearly why they need to collect any particular data and for which purpose and delete them once the employee or student has left the company or university.

A lonely voice among all those calling for more involvement of government or stricter legislation refers to Kant’s dictum “Sapere aude!” to encourage people to use their own intellect rather than rely on the guidance of another. If people decide to divulge their personal data that is their own business and any negative consequences therefore self-inflicted.

## 5 Discussion

As shown in Table 1, most participants are pragmatists who are aware of the pros and cons of sharing personal information. In our case the benefits of sharing personal data include financial benefits, e.g. a reduction of insurance premium or a free news service, as well as intangible rewards such as getting one’s dream job. The costs incurred by data sharing include risks such as potential identity theft or misuse of confidential information. The results of our study do not quite coincide with the findings of the survey conducted by Gerber et al. [8]. Whereas they conclude that the privacy calculus model is among the best predictors for disclosing intention as well as actual disclosure, we found that the possibly gained benefits were unlikely to make people disclose their personal data.

Similarly, our findings fail to confirm the results presented by Engels [5]. She reports that in her survey of 3000 students in Germany, the majority (55%) are not willing to pay for more privacy-friendly services, even in theory, whilst 45 percent would be prepared to pay at least a small amount, e.g. 1 to 10 euros. The findings also reveal the moderating role of people’s need for social interaction and the perceived private nature of both data and setting. Factors such as type of data, setting (e.g. private home) and the purpose of data use also influence people’s risk assessments. Many responses point to the so-called “privacy paradox”, i.e. the phenomenon where people say that they value privacy highly, yet still use their voice assistant provided the (potential) benefits outweigh the risks and/or spend little or no time for measures to protect their privacy (Table 3). This contradiction has been extensively discussed by other researchers such as Solove [6], Winegar and Sunstein [40] and Barth and De Jong [7].

### 5.1 Suggestions regarding privacy-protecting measures on the political-economic level

Actually, most of the suggestions of our digital natives concerning data protection have in theory already been implemented in existing privacy and data protection legislation, such as the General Data Protection Regulation (GDPR) which was designed to regulate the architecture of the personal data economy.

Article 25 of GDPR, for instance, stipulates that digital services comply with the principles of *privacy by design* as well as *privacy by default*. The former is characterized by proactive rather than reactive measures. It anticipates and prevents privacy invasive events before they happen and does not wait for privacy risks to materialize, nor does it offer remedies for resolving privacy infractions once they have occurred − it aims to prevent them from occurring. In short, *privacy by design* comes before-the-fact, not after.

According to the *privacy by default* principle, companies and organisations should by default ensure that personal data is processed with the highest privacy protection. For example, only the data necessary should be processed, it should be stored only for a short period and with limited accessibility so that by default personal data is not made accessible to an indefinite number of persons (“data protection by default”).

These principles are supposed to grant privacy, but apparently participants do not trust tech companies to adhere to them or implement them in a user-friendly way. As pointed out before, many replies reflect feelings of *uneasiness and uncertainty* about how profit-oriented companies would use one’s personal data. Although there are agreements about terms and conditions as well as legal regulations, many participants still feel at the mercy of big tech companies and suspect them of exploiting their data for commercial interests. A recurring theme is the reselling of personal data or its transfer to third parties. Many respondents appear to have been the subject of targeted advertisements based on their search history, for instance. The terms and conditions are not considered very helpful in this respect. On the contrary, they suspect that these are deliberately formulated in a way that makes it hard for users to understand them.

On the other hand, many participants appeal to the government to protect citizens’ personal data. Some call for the creation of a special public authority for protecting citizens’ privacy. At the same time, however, many appear sceptical about the government’s capacity or competence to actually protect their data.

Overall, digital natives appear to espouse a rather critical attitude toward the commercial use of personal data, but are somewhat less critical if the data-collecting body promises to use them for the public interest, for example, to improve health or safety. Moreover, most participants complain about the *lack of transparency* regarding the use of personal data by companies and express concerns about dark patterns and potential surveillance.

### 5.2 Feeling predominantly helpless and powerless

Generally, participants regard their own position vis-à-vis the tech companies as rather weak and vulnerable. Even if a company offers financial incentives or another reward in return for the use of one’s personal data, many are very sceptical about the honesty of such promises. Also, personalised recommendations tend to be viewed with scepticism, at least as far as news are concerned. The filtering of news in line with one’s interests or worldview is seen by many as a *restriction of the freedom of information*. We do not know if they would be equally sceptical with regard to reading or purchasing recommendations based on their search history on Amazon. Whereas in our scenario the filtering was offered to users as an option (and mostly rejected), it is not clear to which extent information is filtered by companies surreptitiously in order to improve the placement of advertising. Similarly, it is not clear according to which criteria Alexa or any other conversational agent selects information, especially if it does not come with a display where the sources of the information are usually visible.

Overall, respondents feel *powerless* in the face of the extensive collection of personal data in all spheres of life. This can be seen in the context of the growing *datafication of human life* [41]. Since the potential negative consequences and risks of general data collection and its aggregation are found to be difficult to gauge, the replies mainly focus on the misuse of confidential data such as health or work-related data. Respondents are less aware of the dangers related to the aggregation of data or data leakages [42]. They are more concerned about any potential loss of confidential information rather than the somewhat diffuse threats posed by today’s technological capability to transform high volumes of data fragments into sensitive personal information [43].

We can conclude that the prevailing attitude with regard to privacy and data protection is characterised by *perceived helplessness and powerlessness*. Given the advances in artificial intelligence and machine learning, such feelings are likely to become even more acute. Data protection regulations by national and supranational organisations seem to be unable to impart the feeling of being in control of the situation.

### 5.3 Limitations of our study

Apart from the small size of our sample, our study suffers from several limitations. One refers to the fact that the students were aware of the importance of privacy and data protection in relation to the use of voice assistant technology because they were introduced to the main findings of the preceding study at the beginning of their course. This may well have resulted in a certain tendency to express serious concerns in this respect. As far as participants’ responses to the scenarios are concerned, attitude and intent are not equal to actual behaviour. Similarly, whilst their responses to the scenarios strongly indicate a willingness to pay for privacy-friendly services, we cannot be sure that they would do so in real life.

Besides, we did not investigate which variables were most relevant for the prediction of attitude, intent or behaviour. We only looked at gender and discipline as potential influencing factors but could not find any differences.

Moreover, it would have been interesting to conduct interviews so as to cast light on some of the surprising lacunae that emerged in the data analysis, e.g. the lack of familiarity with existing or pending legislation or with technological approaches to deal with the challenges posed by new technologies such as conversational agents.

Still, with our study we hope to have presented a first step towards understanding the attitudes, concerns and ideas of digital natives about coping with the privacy challenges associated with conversational agents especially since attempts to practically solve the problem of the privacy paradox are so scarce.

## 6 Conclusions

As we have seen from the responses, even those who are quite aware of potential threats to their privacy and know how to reduce them tend to be resigned to their limited control over their data. Therefore, many students express feelings of resignation, apathy or even cynicism because they believe that privacy violations are inevitable. The matter is further complicated by the so-called “aggregation effect”. People give out bits of data here and there, and each individual disclosure to one particular entity might be relatively innocuous. But when the data is combined, it might reveal a great deal about a person’s habits or preferences.

Thus, risk assessment becomes much more complicated due to recent developments in machine learning. Modern data analytics works via algorithms examining patterns in large quantities of personal data. It is nearly impossible for people to understand the full implications of providing certain pieces of personal data to certain entities. When combined, personal data can reveal facts that people might not want to share. Surprisingly, very few respondents address this problem, or at least only implicitly by expressing their general discomfort with regard to data collection.

As we have seen, there are limits to privacy self-management. Being aware of potential violations of one’s privacy and/or knowing how one can protect oneself against them appears to have a modest effect on behaviour as shown in our own study [1]. Therefore, minimizing behavioural distortion, for example by increasing people’s digital literacy and raising their awareness of potential violations of their privacy, will not cure people’s failure to protect their personal data. It is perfectly rational for people – even without any undue influences on behaviour – to fail to make good assessments of privacy risks and to fail to manage their privacy effectively. Managing one’s privacy is a vast, complex, and never-ending project that does not scale; it becomes virtually impossible to do comprehensively. Privacy regulation often seeks to give people more privacy self-management. Instead, regulation should employ a different strategy, namely focus on regulating the architecture that structures the way information is used, maintained, and transferred (see [6]).

Consequently, privacy-protecting measures have to be taken at regulatory and political levels. However, our respondents appear to be quite unaware of the legislative initiatives at EU level such as the Digital Market Act (DMA) and Digital Services Act (DSA). As pointed out before, most of our digital natives are concerned about dark patterns, which is addressed by the DSA. Though it still needs to be endorsed by the Council and the European parliament it can be expected to address many of the concerns expressed by our respondents. The new rules, which come into force in 2024, include:

– Banning advertising aimed at children or based on sensitive data such as religion, gender, race and political opinions.
– Allowing EU governments to request removal of illegal content, including material that promotes terrorism, child sexual abuse, hate speech and commercial scams.
– Forcing social media platforms to allow users to flag illegal content in an “easy and effective way” so that it can be swiftly removed.
– Online marketplaces like Amazon will need similar systems for suspect products, such as counterfeit sneakers or unsafe toys.

The Digital Markets Act (DMA) also has major implications for the global tech market. The act seeks to prevent the biggest of tech firms from dominating digital markets through the threat of fines or even the possibility of a company breakup. They will also face tighter restrictions on using people’s data for targeted online ads, a primary source of revenue for companies like Google and Facebook. For example, companies would not be allowed to rank their own products or services higher than those of others in online search results or reuse data collected from different services. And a user’s personal data cannot be combined for targeted ads unless “explicit consent” is given. Furthermore, the companies could be forced to hand over data related to their algorithms to regulators and might face a yearly fee of up to 0.05% of worldwide annual revenue to cover the costs of monitoring their compliance.

In many ways these new rules can be compared to capital controls because they regulate which types of data companies can collect, where they can send and store it, or they may include new models of data ownership and governance, e.g. “managing crucial parts of the data economy as public infrastructure” [44]. The fact that *The Economist* [45] should suggest that governments take over parts of the data economy and break up monopolistic firms shows how powerful Big Data has become.

We can conclude that there are regulations and laws already in place or which will come into force soon, which in many ways correspond to and live up to the suggestions and recommendations expressed by the students. However, we note a lack of awareness of their existence which may be due to limited coverage of legal and regulatory affairs in the news channels most commonly used by digital natives. Or it may be due to a general feeling of malaise or scepticism as to the national government’s ability to enforce privacy-related legislation. After all, some of the respondents argue, challenges to privacy require global rather than national approaches or solutions.

Surprisingly, none of them comes up with suggestions about how to design voice assistants in a way to prevent or at least curtail violations of people’s privacy. For example, nobody seems to have heard of Mycroft, an open-source voice assistant, which is private by default and can be integrated into different devices. And the study participants are obviously not familiar with ideas put forward by Seymour and Van Kleek [36] such as refraining from equipping conversational agents with anthropomorphic features that may mislead users to view them as friends. Or with the initiatives of Shorter et al. [46] who investigate provotyping as a design tool for developing voice assistants for privacy, control and transparency.

Finally, though none of the students expresses the sort of radical opinions as espoused by Véliz in her recent essay [37], where she equates digitization with surveillance and calls everything “smart” a spy, a few respondents would agree with her when it comes to defending the analogue world and limiting the purview of the digital:

If we let virtual reality proliferate without limits, surveillance will be equally limitless. If we do not set some ground rules now on what should not be digitized and augmented, the virtual reality will steamroll privacy, and with it, healthy democracies, freedom, and well-being. It is close to midnight [37].
